# Effects of Twelve Sessions of High-Temperature Sauna Baths on Body Composition in Healthy Young Men

**DOI:** 10.3390/ijerph18094458

**Published:** 2021-04-22

**Authors:** Víctor Toro, Jesús Siquier-Coll, Ignacio Bartolomé, Mario Pérez-Quintero, Armando Raimundo, Diego Muñoz, Marcos Maynar-Mariño

**Affiliations:** 1 Department of Physiology, School of Sport Sciences, University of Extremadura, 10003 Cáceres, Spain; vtororom@alumnos.unex.es (V.T.); ignbs.1991@gmail.com (I.B.); marioperezquintero10@gmail.com (M.P.-Q.); mmaynar@unex.es (M.M.-M.); 2 Movement, Brain and Health Research Group (MOBhE), Center of Higher Education Alberta Giménez (Comillas Pontifical University), 07013 Palma of Mallorca, Spain; 3 Comprehensive Health Research Centre (CHRC), Departamento de Desporto e Saúde, Escola de Saúde e Desenvolvimento Humano, Universidade de Évora, Largo dos Colegiais, 7000 Évora, Portugal; ammr@uevora.pt; 4 Department of Didactics of Musical, Plastic and Corporal Expression, School of Teacher Training, University of Extremadura, 10003 Cáceres, Spain; diegomun@unex.es

**Keywords:** hyperthermia, sauna baths, bone mass, muscle mass, DXA

## Abstract

The health benefits of sauna baths are attracting ever-increasing interest. Therefore, the purpose of this study was to evaluate the effects of 12 high-temperature (100 °C) sauna baths on body composition of 23 healthy young men, divided into a control group (CG) and a sauna group (SG). Both groups were initially evaluated by dual-energy X-ray absorptiometry (DXA), after which the SG experienced 12 sessions of sauna baths at high temperatures (100 °C). Initial measurements were carried out after the sauna sessions and after two weeks of decay in both groups. The muscle mass of the right leg (pre vs. decay: 9.50 (5.59) vs. 10.52 (5.15); *p* < 0.05; Δ 1.07%), bone mineral density (pre vs. post: 1.221 (0.35) vs. 1.315 (0.45); *p* < 0.05; Δ 7.7%) and bone mineral content (pre vs. post: 0.470 (0.21) vs. 0.499 (0.22); *p* < 0.05; Δ 6.17%) of the left leg increased in the SG after the sauna baths. It seems that exposure to heat at high temperatures could produce improvements in bone and muscle mass.

## 1. Introduction

There has been growing interest in sauna baths over the last few years [1], and they are increasingly being installed in homes and sports centers [2]. Currently, the uses of the sauna bath are sporting, recreational or rehabilitative [3]. Several studies suggest that sauna baths could provide numerous health benefits [4,5,6], as their use is inversely related to mortality [7].

Heat exposure at high temperatures causes stress in the organism. This stress is detected by peripheral receptors (thermoreceptors and photoreceptors) and central receptors (hypothalamus), reacting to the magnitude of the stress [8]. The organism’s responses are acute and chronic [9]. The acute responses are promoted by the autonomic nervous system, by releasing catecholamines and glucocorticoids [8], eliciting a peripheral blood flow distribution to the skin, increasing sweating in order to remove heat, and provoking, in addition, a rise in heart rate and respiratory rate [10]. The chronic response produces adaptations such as expansion of plasma volume, decreased heart rate, increased sweat rate, reduced mineral excretion through sweating, and improved heat tolerance [9]. These adaptions enhance the cardiovascular system.

However, the influence of sauna baths on body composition has not been studied in depth. It is known that sauna baths elicit a fall in body water due to the rise in sweating [11,12]. Some authors have reported associations between body composition parameters and sauna baths [13,14]. The changes observed in previous studies were related to the loss of body fluids through sweating. Nevertheless, the aforementioned studies carried out short acclimation protocols of 4 sessions or less. Thus, the investigation of the effects of high-temperature sauna baths on body composition is still incomplete. Likewise, to our knowledge, there are no studies which have investigated sauna baths at a temperature above 90 °C.

Heat stress increases the synthesis of heat shock proteins (HSP). These proteins act as molecular chaperones that protect cells by binding to denatured proteins to prevent their aggregation, help transport repair proteins, and transport toxic metabolites for their degradation [15].

Heat stress could promote muscle mass hypertrophy [16]. Yoshiara et al. [17] reported that heat stress activates the kinase B protein/rapamycin target in mammals (Akt/mTOR), a signaling pathway, related to protein synthesis, in rat skeletal muscle. In addition, heat stress attenuates muscle atrophy in immobile persons [18]. Moreover, it has been reported that heat stress could have benefits on lipid metabolism [19]. Similarly, it has been observed that HSP could have a positive effect on bone metabolism [20]. Previous investigations in animals suggest that heat stimulus could promote significant osteogenesis [21,22]; however, no human studies have been found in the scientific literature on this factor. Molecular changes in bone tissues reported in animals has prompted research in humans. Therefore, based on previous research, we hypothesized that the regular use of sauna baths could have positive effects on body composition. Hence, the aim of the present study was to analyze the effect of 12 sessions of sauna baths (100 ± 2 °C) on body composition, evaluated with DEXA, in healthy young men.

## 2. Materials and Methods

### 2.1. Participants

Twenty-three men university students, divided into a sauna group (SG; *n* = 12) and a control group (CG; *n* = 11), voluntarily participated in this study (Table 1). Participants were randomly assigned to each group using a mobile application. All the participants were informed about the purpose of the study and signed a consent form before enrolling. The protocol was reviewed and approved by the Biomedical Ethics Committee of the University of Extremadura (Spain) (Registration code: 33/2020) following the guidelines of the Helsinki declaration of ethics, updated at the World Medical Assembly in Fortaleza (2013), for research involving human subjects. A code was assigned to each participant for the collection and treatment of the samples to maintain their anonymity. To be included in the study, the participants had to comply with the following inclusion criteria: be a healthy man, not have taken any supplementation, medication or over-the-counter medication, drug or alcohol in the previous two weeks and not to change their nutritional and physical activity habits during the study.

### 2.2. Experimental Design

The experimental phase lasted 6 weeks. Initially, anthropometric and body composition measurements were taken. Subsequently, 12 sessions of heat exposure were administered to the SG for 4 weeks (3 procedures per week). All participants underwent the same procedure during the 12 sessions. After exposure, the initial tests were repeated, and again after 2 weeks of decay in both groups.

### 2.3. Sauna Baths

This protocol was similar to the one carried out by Siquier-Coll et al. [23]. The sessions consisted of 5 sets of 10-min exposure to heat at high temperatures (100 °C; 20–30% relative humidity) in a sauna (Harvia C105S Logix Combi Control; 3–15 W; Finland) with 5 min recovery at laboratory temperature (22 °C; 40–50% RH) between sets. The sessions were performed at the same time of day for each participant. The participants were allowed to drink water ‘ad libitum’ (300–500 mL) during the heat exposure sessions.

### 2.4. Health Security Protocol

Previously to the experimental period, all participants had been examined by a physician to avoid any cases of illness or contraindications to participating in the study. Core temperature (Tc), measured in the buccal mucosa, and skin temperature (Tsk), measured on the forehead in triplicate, were monitored using an infrared thermometer [TAT 5000 “Exergen Temporal Scanner” (Corp., Watertown, MA, USA)] at the beginning and end of each set. Furthermore, heart activity was monitored in real time in the tests by mean of pulsometers (Polar M Vantage) during the sessions and recovery times. Systolic and diastolic blood pressure was measured with an electronic blood pressure monitor (OMRON M6 Comfort) with the subject seated at the beginning and at the end of each set. No diseases were reported during the study.

### 2.5. Physical Activity Assessment

Physical activity was assessed using the International Physical Activity-Short Form Questionnaire (IPAQ-SF; Spanish version) [23]. The questionnaire consists of questions on the frequency and duration of vigorous-intensity activity, moderate-intensity activity, and walking activity. In addition, the IPAQ-SF contained an item indicating the time spent in sedentary activity. The questionnaire was scored using established methods published on the IPAQ website (www.ipaq.ki.se, accessed on 13 November 2019). A researcher helped the participants to answer. The time spent on vigorous, moderate, and walking activity was weighted by the energy spent for these activity categories, to produce the total MET-minutes of physical activity per week. The questionnaire was administered at the beginning, after the 12 sauna bath sessions and during the last week of the decay.

### 2.6. Nutritional Intake Assessment

All participants completed a nutritional survey at the start of the study, after the 12 sessions, and the last week of the study to ensure that they were following a similar diet. The survey consisted of a 4-day daily nutritional record on three pre-assigned business days and one weekend day. The participants individually indicated the type, frequency and quantity (in grams) of each food consumed each day. After this, the nutritional composition of their diets was evaluated using different food composition tables [24].

### 2.7. Assessment of Body Composition

Height was measured using a SECA stadiometer. Bodyweight was estimated by electric bioimpedance BF-350 (Tanita Corp. Amsterdam the Netherlands). Body fat mass (FM), percentage of fat (%FM), bone mineral density (BMD), bone mineral content (BMC), lean body mass (LBM), as well as fat-free mass (FFM) were assessed with dual-energy X-ray absorptiometry (DXA—Hologic QDR, Hologic, Inc., Bedford, MA, USA). Standard positioning, with subjects lying supine, was used to scan the full body. The same experienced technician performed all the scans.

### 2.8. Statistical Analysis

The software IBM SPSS Statistics 20.0 for Windows (IBM, Armonk, NY, USA) was used to carry out the statistical analysis. The statistical evaluation consisted of an initial Shapiro–Wilk test to examine the distribution of the variables and Levene’s test to examine variance homogeneity. The Wilcoxon test for intra-group differences and the Mann Whitney U test for inter-group differences were used for data analysis. Effect size (ES) was calculated according to Tomczack and Tomczack [25]. ES of 0.2, 0.4, and 0.8 were considered small, moderate, and large, respectively [26]. Power analysis was conducted with G*Power 3.1.9.7 software. A *p* < 0.05 was considered statistically significant. The results are expressed as medians and range.

## 3. Results

The results of the study are presented below. Power analysis showed that with an alpha set at *p* < 0.05 the achieved power and effect size in the present study were 0.527 and 0.172, respectively. Table 2 shows the data on the physical activity performed and the sitting time of each group during the study. No differences in physical activity and sitting time were observed during the study.

Macronutrient intake data for both groups are presented in Table 3. No significant differences were found in either group.

### Body Composition

Table 4 and Table 5 show the evolution of body composition parameters during the study in both groups. In Table 4, an increase in the muscle mass of the right leg was observed at the end of the study in the SG (*p* < 0.05). In contrast, at the beginning of the study and at the end of the sauna baths, the muscle mass of the right leg was higher in the CG (*p* < 0.05).

Table 5 shows increases in BMC and BMD in the left leg in the SG (*p* < 0.05). Likewise, BMC and BMD values of the left leg were higher in the SG compared to the CG (*p* < 0.05) after exposure to high temperatures.

## 4. Discussion

The aim of this study was to analyze the effect of 12 sauna bath sessions (100 ± 2 °C) on the body composition of healthy young men. Conversely to our hypothesis, there were no changes in the fat parameters; however, some increases in muscle and bone parameters were observed in the SG. To our knowledge, there is no research that has analyzed the effect of sauna baths at such high temperatures on humans. Likewise, we have found no studies that analyze the influence of sauna baths on bone tissue in humans. This is a preliminary study that aimed to observe the changes produced by sauna baths at high temperatures in body composition. Thus, the participants were healthy young men.

There were no differences between the two groups in either physical activity or nutritional intake, as seen in the results in Table 1 and Table 2. In addition, as mentioned in the inclusion criteria, the subjects did not change their lifestyles during the research. Therefore, the changes observed could be because of the 12 sessions of exposure to heat. It should be noted that despite the high temperatures, all participants completed the experimental phase. Regarding effect size, there was a moderate effect size only for those parameters that showed significant differences. However, due to the small sample size, the results obtained should be assessed with caution.

The studies that have observed the effects of sauna baths on body composition have been carried out on sedentary and overweight people [13,14,27]. In addition, the temperature used in the studies mentioned above was lower than in the present study and involved fewer sessions. Podstawski et al. [14] reported that overweight subjects lost more body weight compared to normal-weight subjects. Similarly, Podstawski et al. [27] reported that higher body mass leads to a significant increase in sauna-bath-induced body mass loss, stating that body mass had a significant influence on body mass loss. Moreover, Podstawski et al. [14] observed that energy expenditure and body mass loss were correlated with anthropometric parameters and body composition parameters (percentage of body fat, body fat mass, and visceral fat level). Conversely, Sakurai et al. [28] observed that sauna baths did not produce changes in body composition parameters in overweight people. However, exercise combined with diet significantly reduced the percentage of fat and weight. In our study, the subjects were physically active (reporting 7 days per week combining walking or moderate or high intensity activities with a minimum of 3000 MET-min/week; or vigorous activity at least 3 days per week of at least 1500 MET-min/week.) [29], with a normal BMI and low fat percentage, being subjects of different characteristics to those of the studies mentioned. Gryka et al. [30] reported no change in body composition after 10 sessions of sauna baths at 90 °C in physically active men. The baseline data of the participants in the above-mentioned studies and our research is a factor to be considered when interpreting the results. Participants with a normal BMI (20–24.9 kg/m^2^) [27], such as the participants in our study, would have more difficulty in reducing weight and fat percentage compared to populations with a high BMI [28]. Therefore, we believe that there were no changes in weight and fat tissue due to the low initial values of the subjects. The loss of body mass associated with sauna bathing is related to the body’s fluid loss. Sweat volume during sauna bathing is estimated at 0.6 to 1.0 kg/h, and rising temperature and humidity increase sweating, although individual responses could fluctuate. [13].

In the present study, an increase in muscle mass in the right leg was observed after the sauna baths. It has previously been shown that muscle warming before and during low-intensity strength exercise causes an increase in the cross-section of the muscle [31]. However, other authors did not observe significant changes following local warming in the muscle [32]. In contrast to our study, previous research used a local warm-up rather than a full-body warm-up. Glazachez et al. [33] and Zapara et al. [34] found no change in fat mass but an increase in muscle mass after the application of the heat sessions (65–80 °C), as we did. However, the above studies applied heat using infrared capsules and obtained changes after 24 sessions instead of 12. Increased expression of HSP 72 and 24 and intracellular signaling of protein synthesis through heat stress have been postulated as the main factors contributing to increased muscle hypertrophy [17,35]. It is known that heat stress increases phosphorylation of signaling kinases (Akt, mTOR, p70S6K [ribosomal p70 S6 kinase]) which regulate muscle protein synthesis [17,36]. Another beneficial effect of heat stress that could influence muscle mass is an increase in blood flow to supply nutrients and anabolic hormones to the muscle [37]. We believe that unlike the previous studies discussed, whole-body exposure to heat would further increase the signaling of muscle protein synthesis.

As a study novelty, increases in BMC and BMD of the left leg were observed in the SG. Human evidence on this topic is scarce. Ota et al. [21] observed significant increases in osteogenesis and bone formation area in rats and rabbits after 15 min of hyperthermia at 45 °C, 3 times per week in rats and once per week in rabbits. Similarly, Shuit and Scutt [38] concluded that treatment with mild heat induces proliferation and differentiation of osteoprogenitor cells. Kajiya et al. [39] showed that daily heat treatment at 42 °C for 10 min increases alkaline phosphatase activity. Differing from our study, all the aforementioned studies have been conducted on animals or on isolated human cells. In humans, we have not found any studies that discuss this topic. The mechanisms of heat-stress-enhanced osteogenesis have not been clearly elucidated. The involvement of HSP70 in promoting osteogenesis has been reported [40]. HSP70 enhances alkaline phosphatase activity and promotes bone mineralization. Similarly, HSP70 may significantly raise the expression of genes related to bone tissue [21]. Other HSP such as HSP60 could also influence bone metabolism [20]. The involvement of HSP should be further explored. As another possible mechanism, Li et al., reported that heat stress promoted angiogenesis, increasing osteoconduction and contributing to osteogenesis [41]. We think that the results obtained in BMD and BMC in the left leg could be due to less involvement during the daily life of the participants. The present study shows another alternative to maintaining or increasing BMD or BMC passively, similar to that observed in muscle tissue [18]. Further studies are needed to clarify the results.

Concerning decay, it has been reported that the benefits of heat acclimation can last up to three weeks longer in the cardiovascular system [42], thermoregulatory system [43], physical performance [44] and sweating rate [45]. To our knowledge, there are no investigations on the durability of heat acclimation in the locomotor system. Our study shows that the benefits obtained in muscle mass are better even after two weeks of decay than after 12 sauna sessions. This would indicate that repeated heat exposure could still produce benefits in muscle mass after two weeks without exposure or the enhancement could have been produced by super-compensation. Elsewhere, BMD and BMC increased in the left leg after the 12 sauna baths, but the benefits declined after 2 weeks of decay. Therefore, gains in bone mass are affected after two weeks of decay. Further research is needed on the effect of heat acclimation and decay on the locomotor system.

In the current study, some limitations can be highlighted: (1) the small sample size, (2) the participants were physically active men with normal BMI, which could influence changes in body composition and (3) no data was recorded for the dominant legs of the participants, a fact that could have influenced the results obtained in BMD and BMC. Future studies on this topic should focus on sedentary people who are overweight or have bone diseases, due to the benefits observed.

## 5. Conclusions

Twelve sessions of sauna baths at high temperatures (100 ± 2 °C) do not produce changes in fat parameters. However, in view of the results obtained in the present study, they could perhaps influence muscle and bone parameters. Our study shows that sauna baths could be a promising treatment option for diseases associated with bone mineral alterations and sarcopenia. Further studies in humans are needed to confirm these encouraging results.

## Figures and Tables

**Table 1:** Participants’ characteristics.

	SG (*n* = 12)	CG (*n* = 11)
Age (years)	19.7 ± 1.5	20.3 ± 2.1
Weight (kg)	66.0 ± 9.7	66.4 ± 7.4
Height (m)	1.7 ± 0.1	1.7 ± 0.1
BMI (kg/m^2^)	22.0 ± 1.9	23.4 ± 1.8

SG: sauna group; CG: control group. Expressed in means ± standard deviation.

**Table 2:** Physical activity and sitting time of each group at the beginning, after 12 sessions of sauna and after two weeks of decay.

		Beginning	After 12 Sessions	Decay	Differences	*p*
Total physical activity(Met-min/week)	SG	1523.5 (324.1)	1645.6 (367.4)	1671.2 (341.6)	Beginning vs. after 12 sessions	0.418
					Beginning vs. Decay	0.376
					After 12 sessions vs. Decay	0.612
	CG	1478.3 (402.2)	1511.6 (429.8)	1532.2 (422.1)	Beginning vs. after 12 sessions	0.537
					Beginning vs. Decay	0.478
					After 12 sessions vs. Decay	0.490
Sitting time (h/week)	SG	6.21 (1.1)	6.0 (1.2)	6.1 (1.3)	Beginning vs. after 12 sessions	0.311
					Beginning vs. Decay	0.297
					After 12 sessions vs. Decay	0.566
	CG	6.1 (1.4)	6.3 (1.4)	6.2 (1.2)	Beginning vs. after 12 sessions	0.325
					Beginning vs. Decay	0.502
					After 12 sessions vs. Decay	0.382

SG: sauna group; CG: control group.

**Table 3:** Nutritional intake of macronutrients in both groups during the study.

		Beginning	After 12 Sessions	Decay	Differences	*p*
Energy (Kcal/day)	SG	1584.2 (326.4)	1476.3 (367.1)	1561.3 (356.6)	Beginning vs. after 12 sessions	0.436
					Beginning vs. Decay	0.503
					After 12 sessions vs. Decay	0.310
	CG	1634.4 (294.6)	1574.1 (268.2)	1780.4 (283.1)	Beginning vs. after 12 sessions	0.421
					Beginning vs. Decay	0.545
					After 12 sessions vs. Decay	0.561
Proteins (g/day)	SG	78.3 (18.4)	69.2 (15.6)	76.4 (16.7)	Beginning vs. after 12 sessions	0.280
					Beginning vs. Decay	0.514
					After 12 sessions vs. Decay	0.389
	CG	75.1 (14.2)	70.4 (15.7)	76.3 (16.4)	Beginning vs. after 12 sessions	0.312
					Beginning vs. Decay	0.678
					After 12 sessions vs. Decay	0.221
Lipids (g/day)	SG	57.3 (18.5)	48.4 (16.8)	49.7 (17.3)	Beginning vs. after 12 sessions	0.158
					Beginning vs. Decay	0.124
					After 12 sessions vs. Decay	0.638
	CG	61.4 (15.4)	52.4 (16.6)	50.8 (15.3)	Beginning vs. after 12 sessions	0.196
					Beginning vs. Decay	0.102
					After 12 sessions vs. Decay	0.534
HCO (g/day)	SG	204.4 (53.1)	185.3 (65.21)	183.7 (61.23)	Beginning vs. after 12 sessions	0.268
					Beginning vs. Decay	0.221
					After 12 sessions vs. Decay	0.554
	CG	198.8 (26.1)	193.7 (25.7)	197.8 (26.3)	Beginning vs. after 12 sessions	0.452
					Beginning vs. Decay	0.698
					After 12 sessions vs. Decay	0.412

SG: sauna group; CG: control group; HCO: carbohydrates.

**Table 4:** Weight and percentage of muscle mass and fat mass in both groups.

		Beginning	After 12 sessions	Decay		*p*	ES
Weight (Kg)	SG	64.2 (29.0)	64.1 (31.9)	64.4 (29.0)	Beginning vs. after 12 sessions	0.530	0.13
					Beginning vs. Decay	0.628	0.10
					After 12 sessions vs. Decay	0.782	0.06
	CG	65.1 (28.3)	66.3 (29.5)	66.1 (28.2)	Beginning vs. after 12 sessions	0.518	0.12
					Beginning vs. Decay	0.410	0.22
					After 12 sessions vs. Decay	0.612	0.09
Fat mass (kg)	SG	9.6 (1.6)	9.8 (1.5)	9.9 (1.6)	Beginning vs. after 12 sessions	0.480	0.20
					Beginning vs. Decay	0.454	0.22
					After 12 sessions vs. Decay	0.608	0.09
	CG	10.4 (2.1)	11.7 (2.7)	12.1 (2.3)	Beginning vs. after 12 sessions	0.281	0.31
					Beginning vs. Decay	0.207	0.35
					After 12 sessions vs. Decay	0.382	0.26
Fat mass without head (Kg)	SG	8.6 (1.6)	8.8 (1.5)	8.9 (1.6)	Beginning vs. after 12 sessions	0.520	0.10
					Beginning vs. Decay	0.543	0.13
					After 12 sessions vs. Decay	0.618	0.07
	CG	9.2 (1.4)	10.0 (1.5)	10.3 (1.4)	Beginning vs. after 12 sessions	0.378	0.17
					Beginning vs. Decay	0.232	0.33
					After 12 sessions vs. Decay	0.413	0.24
Fat mass (%)	SG	14.5 (1.9)	14.5 (2.0)	14.0 (2.1)	Beginning vs. after 12 sessions	0.814	0.04
					Beginning vs. Decay	0.875	0.03
					After 12 sessions vs. Decay	0.695	0.06
	CG	15.7 (2.4)	16.4 (2.1)	16.0 (2.7)	Beginning vs. after 12 sessions	0.421	0.23
					Beginning vs. Decay	0.478	0.20
					After 12 sessions vs. Decay	0.402	0.24
Muscle mass (Kg)	SG	55.6 (32.8)	55.7 (30.8)	56.2 (30.6)	Beginning vs. after 12 sessions	0.388	0.25
					Beginning vs. Decay	0.286	0.33
					After 12 sessions vs. Decay	0.458	0.21
	CG	52.2 (29.6)	51.8 (28.4)	52.4 (28.2)	Beginning vs. after 12 sessions	0.389	0.25
					Beginning vs. Decay	0.411	0.22
					After 12 sessions vs. Decay	0.521	0.10
Right leg muscle mass (%)	SG	9.5 (5.6)	9.5 (5.4)	10.5 (5.1)	Beginning vs. after 12 sessions	0.554	0.09
					Beginning vs. Decay	0.035	0.64
					After 12 sessions vs. Decay	0.042	0.60
	CG	10.6 (4.7) *	10.5 (4.3) *	10.2 (4.2)	Beginning vs. after 12 sessions	0.578	0.09
					Beginning vs. Decay	0.632	0.09
					After 12 sessions vs. Decay	0.591	0.08
Right leg fat mass (Kg)	SG	1.4 (0.6)	1.5 (0.5)	1.5 (0.5)	Beginning vs. after 12 sessions	0.533	0.09
					Beginning vs. Decay	0.737	0.05
					After 12 sessions vs. DA	0.695	0.09
	CG	1.8 (0.1)	1.7 (0.3)	1.7 (0.5)	Beginning vs. after 12 sessions	0.732	0.06
					Beginning vs. Decay	0.490	0.19
					After 12 sessions vs. Decay	0.412	0.12
Left leg muscle mass (%)	SG	7.7 (1.1)	7.8 (1.1)	7.8 (1.0)	Beginning vs. after 12 sessions	0.308	0.29
					Beginning vs. Decay	0.291	0.30
					After 12 sessions vs. Decay	0.621	0.09
	CG	8.1 (1.0)	8.1 (1.1)	8.1 (1.0)	Beginning vs. after 12 sessions	0.653	0.08
					Beginning vs. Decay	0.734	0.06
					After 12 sessions vs. Decay	0.658	0.08
Left leg fat mass (kg)	SG	1.9 (0.3)	1.8 (0.3)	1.8 (0.3)	Beginning vs. after 12 sessions	0.533	0.07
					Beginning vs. Decay	0.581	0.07
					After 12 sessions vs. Decay	0.795	0.05
	CG	1.8 (0.3)	1.9 (0.3)	1.9 (0.3)	Beginning vs. after 12 sessions	0.635	0.08
					Beginning vs. Decay	0.789	0.07
					After 12 sessions vs. Decay	0.712	0.08

SG: sauna group; CG: control group. * Differences between groups (*p* < 0.05).

**Table 5:** Changes in bone parameters during the study.

		Beginning	After 12 Sessions	Decay		*p*	ES
BMC total (kg)	SG	2.381 (1.05)	2.352 (1.10)	2.385 (0.98)	Beginning vs. after 12 sessions	0.530	0.15
					Beginning vs. Decay	0.399	0.25
					After 12 sessions vs. Decay	0.336	0.26
	CG	2.405 (0.99)	2.386 (1.01)	2.391 (1.02)	Beginning vs. after 12 sessions	0.645	0.08
					Beginning vs. Decay	0.594	0.09
					After 12 sessions vs. Decay	0.582	0.10
BMC without head (kg)	SG	1.900 (0.10)	1.911 (0.10)	1.898 (0.10)	Beginning vs. after 12 sessions	0.688	0.07
					Beginning vs. Decay	0.754	0.06
					After 12 sessions vs. Decay	0.639	0.07
	CG	1.895 (0.09)	1.902 (0.10)	1.905 (0.11)	Beginning vs. after 12 sessions	0.848	0.04
					Beginning vs. Decay	0.743	0.06
					After 12 sessions vs. Decay	0.652	0.08
BMC left leg (kg)	SG	0.470 (0.21)	0.499 (0.22)	0.484 (0.20)	Beginning vs. after 12 sessions	0.033	0.65
					Beginning vs. Decay	0.158	0.38
					After 12 sessions vs. Decay	0.447	0.21
	CG	0.469 (0.23)	0.470 (0.21) *	0.476 (0.21)	Beginning vs. after 12 sessions	0.703	0.07
					Beginning vs. Decay	0.734	0.06
					After 12 sessions vs. Decay	0.612	0.08
BMC right leg (kg)	SG	0.474 (0.27)	0.465 (0.26)	0.467 (0.28)	Beginning vs. after 12 sessions	0.378	0.25
					Beginning vs. Decay	0.347	0.28
					After 12 sessions vs. Decay	0.738	0.07
	CG	0.466 (0.24)	0.468 (0.26)	0.475 (0.25)	Beginning vs. after 12 sessions	0.517	0.11
					Beginning vs. Decay	0.689	0.07
					After 12 sessions vs. Decay	0.702	0.08
Total BMD (g/cm^2^)	SG	1.143 (0.28)	1.150 (0.34)	1.134 (0.34)	Beginning vs. after 12 sessions	0.495	0.18
					Beginning vs. Decay	0.608	0.09
					After 12 sessions vs. Decay	0.582	0.10
	CG	1.146 (0.23)	1.143 (0.22)	1.144 (0.25)	Beginning vs. after 12 sessions	0.537	0.11
					Beginning vs. Decay	0.639	0.08
					After 12 sessions vs. Decay	0.651	0.08
BMD without head(g/cm^2^)	SG	0.996 (0.31)	1.015 (0.33)	0.997 (0.34)	Beginning vs. after 12 sessions	0.475	0.20
					Beginning vs. Decay	0.347	0.27
					After 12 sessions vs. Decay	0.533	0.12
	CG	1.001 (0.28)	1.009 (0.26)	1.001 (0.31)	Beginning vs. after 12 sessions	0.773	0.06
					Beginning vs. Decay	0.602	0.10
					After 12 sessions vs. Decay	0.658	0.07
Left leg BMD (g/cm^2^)	SG	1.221 (0.35)	1.315 (0.45)	1.273 (0.49)	Beginning vs. after 12 sessions	0.045	0.57
					Beginning vs. Decay	0.483	0.19
					After 12 sessions vs. DA	0.206	0.34
	CG	1.242 (0.31)	1.231 (0.32) *	1.261 (0.30)	Beginning vs. after 12 sessions	0.523	0.14
					Beginning vs. Decay	0.812	0.03
					After 12 sessions vs. Decay	0.452	0.21
Right leg BMD (g/cm^2^)	SG	1.226 (0.45)	1.226 (0.50)	1.224 (0.43)	Beginning vs. after 12 sessions	0.814	0.04
					Beginning vs. Decay	0.317	0.26
					After 12 sessions vs. Decay	0.382	0.17
	CG	1.234 (0.42)	1.242 (0.41)	1.245 (0.41)	Beginning vs. after 12 sessions	0.615	0.09
					Beginning vs. Decay	0.587	0.08
					After 12 sessions vs. Decay	0.801	0.04

BMC = Bone Mineral Content: BMD = Bone Mineral Density. * Differences between groups (*p* < 0.05).
